# Importance of oral health in mental health disorders: An updated review

**DOI:** 10.1016/j.jobcr.2023.06.003

**Published:** 2023-06-19

**Authors:** Hans Erling Skallevold, Nabin Rokaya, Natthamet Wongsirichat, Dinesh Rokaya

**Affiliations:** a Department of Oral and Maxillofacial Surgery, Faculty of Dentistry, Chulalongkorn University, Bangkok, 10330, Thailand; b Humla Hospital District, Huma, 21000, Nepal; c Faculty of Dentistry, Bangkok Thonburi University, 16/10 Taweewatana, Bangkok, 10170, Thailand; d Department of Clinical Dentistry, Walailak University International College of Dentistry, Bangkok, 10400, Thailand

**Keywords:** Psycho-disorders, Mental health, Oral health, Oral health approaches

## Abstract

**Background:**

Mental disorders are indeed an expanding threat, which requires raised awareness, education, prevention, and treatment initiatives nationally and globally. This review presents an updated review on the relationships between oral health and mental health disorders and the importance of oral health in mental health disorders.

**Method:**

A literature search was done regarding mental disorders and oral health approaches in Google Scholar and PubMed from the year 1995 until 2023. All the English-language papers were evaluated based on the inclusion criteria. Publications included original research papers, review articles and book chapters.

**Results:**

Common mental disorders include depression, anxiety, bipolar disorder, Schizophrenia, dementia, and alcohol and drug use disorders. The interplay of oral health and mental disorders involves dysregulated microbiome, translocated bacteria, and systemic inflammation, among others.

**Conclusion:**

There is a complex relationship between mental disorders and oral diseases. Various oral health problems are associated with mental health problems. The interplay of oral health and mental disorders involves dysregulated microbiome, translocated bacteria, and systemic inflammation, among others. Mental health nurses including physicians and dental professionals should be involved in the oral health care of mental health disorder patients. Therefore, multidisciplinary should be involved in the care of mental health disorders, and they should consider oral health care as an essential part of their care for patients with mental health disorders. Future investigations should strive to elucidate the exact biological relationships, to develop new directions for treatment.

## Highlights

• Mental health problems are increasing as the consequences of the covid-19 pandemic.
• Various oral health problems are associated with mental health problems.
• Oral health and mental disorders involve dysregulated microbiome and inflammation.

## Graphical abstract

**Figure:**
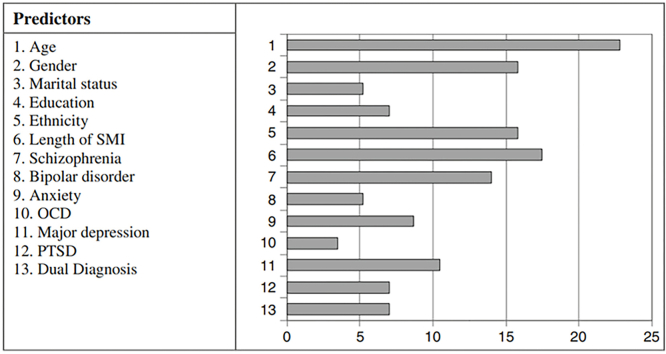

## 1 Introduction

Recently, the importance of mental health has been highlighted in light of the consequences of the covid-19 pandemic.^1^ The sudden change of living, involving quarantine and social distancing during the pandemic may have contributed to the growing number of reports of mental health problems.^2^ The World Health Organization (WHO) promotes mental health as an integral part of general health, as they define it as the state of “well-being where an individual realizes his or her abilities, can cope with the normal stresses of life, can work productively and can make a contribution to his or her community”.^3^ Mental health is necessary for a well-functioning individual and community, through interaction and development. When an individual's mental state exhibits certain behavioral or psychological patterns; a mental disorder may be diagnosed based on criteria in The Diagnostic and Statistical Manual of Mental Disorders, 5th edition, the DSM-5 for short.^4^ The term mental disorder, influences several aspects of life and is sometimes interchangeably used with psychological and psychiatric disorders. Prior to the pandemic, the number of persons with a mental disorder was counted to be around 792 million globally, this prevalence has surged by about 25%.^2^^,^^5^^,^^6^

Several socio-economic aspects, typically unemployment, social isolation, and poverty, impact mental disorders.^7^^,^^8^ A number of modifications in people's health behaviors have occurred as an effect of the covid-19 pandemic, such as a rise in snacking, tobacco and alcohol consumption, and decreased physical activity.^9^ When negative health behaviors combine with stressors such as fear of infections, lack of supplies and information, or financial loss (these are relevant for the covid-19 pandemic), oral health may deteriorate.^10^ As mental disorders are on the rise, one may expect an increase in anti-psychotics and depressants. These medications involve the risk of adverse effects, such as bruxism and xerostomia, which adversely affect the oral.^11^, ^12^, ^13^, ^14^ The notion that fear of infection and social interaction, associated with the pandemic, may hinder access to dental, has been reported.^15^ Worsened anxiety or phobia of dental treatment can result from non-attendance,^16^^,^^17^ making patients exclusively seek dental care during emergencies.^18^

The pandemic's impact on mental health is distressing,^19^ however, this may just be among the first of several sequelae to appear. For example, the pandemic may have fueled anxiety through elevated use and addiction to social media,^20^, ^21^, ^22^, ^23^ social media may also influence the perception of dental treatment.^24^^,^^25^ Indeed, about 3.5 billion individuals have untreated oral conditions,^26^ and the number may grow with the growing prevalence of mental disorders and as a consequence of the pandemic. Mental disorders and oral health's reciprocal influence is generally neglected and little-known issue.^27^ However, this issue is highly relevant following the pandemic and needs to be brought to awareness by health professionals to guide policymaking. This review presents an updated review on the relationships between oral health and mental health disorders and the importance of oral health in mental health disorders.

## 2 Method

A literature search was done regarding mental disorders and oral health approaches in Google Scholar and PubMed from the year 1995 until 2023. All the English-language papers were evaluated based on the inclusion criteria. Publications included original research papers, review articles, and book chapters.

## 3 Psycho-disorders/mental disorders definition, prevalence, types, symptoms, or habits

In 2017, about 792 million individuals were diagnosed with a mental disorder on a global scale, with females as the dominating gender.^28^ Mental disorders encompass a diverse spectrum, as summarized in Table 1. A mental disorder involves a person's behavioral or psychological patterns; based on criteria in the DSM-5.^4^ Most encountered disorders are depression and anxiety, affecting around 3.8% of the world population. Recently, the global prevalence of depression and anxiety, in adolescents was estimated to be 25–31%.^29^ More than half of middle‐ and high‐income countries' populations are expected to suffer from one, or more, mental disorders during their lives.^30^ Poor mental health is a growing burden globally, in the span from 1990 to 2019, the global proportion of disability-adjusted life-years ascribed to mental disorders went from 3.1 to 4.9%.^31^ This has a profound impact on the world economy, as costs involve more than direct costs such as medication and hospitalization,^32^ but also income losses because of lost production because of missing work or swift retirement.^32^^,^^33^ Such indirect costs did make up 1.7 trillion USD in 2010, while the total costs totaled 2.5 trillion, these numbers are only expected to rise with the growing prevalence.^33^ The projected lost economic output globally, from 2011 to 2030, is estimated to reach 16.3 trillion USD, which surpasses that of cancer and is similar to cardiovascular diseases.^33^ Mental disorders are indeed an expanding threat, which requires raised awareness, education, prevention, and treatment initiatives nationally and globally.^31^ Common metal disorders include depression, anxiety, bipolar disorder, Schizophrenia, dementia, and alcohol and drug use disorders (Table 2).

**Table 1:** Overview of the global prevalence in 2017 of common mental disorders. Adapted with permission from Dattani et al.^28^

Disorder	Share of the global population with the disorder (2017) [difference across countries]	Number of people with the disorder (2017)	Share of males: females with the disorder (2017)
Any mental health disorder	10.7%	792 million	9.3% males 11.9% females
Depression	3.4% [2–6%]	264 million	2.7% males 4.1% females
Anxiety disorders	3.8% [2.5–7%]	284 million	2.8% males 4.7% females
Bipolar disorder	0.6% [0.3–1.2%]	46 million	0.55% males 0.65% females
Eating disorders (clinical anorexia & bulimia)	0.2% [0.1–1%]	16 million	0.13% males 0.29% females
Schizophrenia	0.3% [0.2–0.4%]	20 million	0.26% males 0.25% females
Any mental or substance use disorder	13% [11–18%]	970 million	12.6% males 13.3% females
Alcohol use disorder	1.4% [0.5–5%]	107 million	2% males 0.8% females
Drug use disorder (excluding alcohol)	0.9% [0.4–3.5%]	71 million	1.3% males 0.6% females

**Table 2:** Common mental health disorders and their common symptoms.

Common metaldisorders	Common Sympoms
Depression	Symptoms mostly involve sadness and loss of interest or pleasure.
Anxiety	Anxiety sets off in presence of an overactivation of a recognized threat or erroneous danger assessment, leading to an excessive and unfitting fight-or-flight reaction
Bipolar disorder	Usually comprises manic and depressive episodes, with periods of normal mood in-between. High-speed speech, elevated self-esteem, and reduced need for sleep, typically characterize a manic episode
Schizophrenia and other psychoses	Schizophrenia is a typical psychosis, characterized by distorted thinking, perception, emotions, and behavior, with hallucinations of auditory and visual types
Dementia	Findings of cognitive decline compared to a former time point, while the person remains independent and well-functioning in daily life, is diagnosed with mild cognitive impairment

### 3.1 Depression

Depression is, together with anxiety, the most common mental disorder and one of the major reasons for disability.^28^ Symptoms mostly involve sadness and loss of interest or pleasure.^34^ Compromised social function, impacting education or work, is common and involves a significant risk of suicide.^35^

### 3.2 Anxiety

Anxiety sets off in the presence of an overactivation of a recognized threat or erroneous danger assessment, leading to an excessive and unfitting fight-or-flight reaction.^36^ Such irrational fear responses give off a wide scope of symptoms; fear of losing control or of death, diminished concentration, elevated confusion, impaired memory, tachycardia, shortness of breath, chest pains, syncope, and so on. People with anxiety tend to avoid triggering situations.^37^ Several subtypes of anxiety exist, a common one is dental anxiety or dental fear. The more severe version is known as dental phobia, or odontophobia; an extremely irrational fear or aversion to dental-related situations.^38^ A recent meta-analysis estimated the global prevalence of dental anxiety in children and adolescents to be 23.9%. Whereas 36.5% of preschoolers, 25.8% of school children, and 13.3% of adolescents, reported dental anxiety.^39^ Dental anxiety is common with a 36% prevalence, while dental phobia affects a smaller proportion of the population, about 10% globally.^40^ Dental anxiety and phobia impact oral health and general well-being negatively and make necessary dental treatment challenging. For these patients, it is vital for the practitioner to offer a safe environment and trust, and use an array of approaches; information, show-do-tell, coping skills, cognitive behavior therapy, preventive and minimally invasive treatment.^41^ Specialists, both medical and dental, or psychologists, with or without sedation with nitrous oxide or benzodiazepines, or even general anesthesia, may be necessary in select cases.^38^

### 3.3 Bipolar disorder

Close to 46 million people worldwide^28^ are affected by bipolar disorder. Usually, it comprises manic and depressive episodes, with periods of normal mood in-between. High-speed speech, elevated self-esteem, and reduced need for sleep, typically characterize a manic episode. Bipolar disorder impacts several aspects, such as increased mortality and disability, and diminished quality of life.^42^

### 3.4 Schizophrenia and other psychoses

Schizophrenia is a typical psychosis, characterized by distorted thinking, perception, emotions, and behavior, with hallucinations of auditory and visual types. Untreated schizophrenia is not compatible with a normal function or role in society, however, effective treatment and support can re-integrate the person into society with a productive life.^43^

### 3.5 Dementia

Objective findings of cognitive decline compared to a former time point, while the person remains independent and well-functioning in daily life, is diagnosed with mild cognitive impairment. The diagnosis of dementia requires supplemental findings of significant difficulties in daily life, and that negatively influence the person's independence.^44^ That is the consequence of impaired cognitive functions such as memory, motivation, thinking, orientation, emotional control, and communication, among others. Dementia is mostly the product of Alzheimer's disease or stroke. The most apparent and significant risk factor is age.^45^^,^^46^

The global scale prevalence is projected to grow from 57 million individuals to 152 million by 2050,^47^ this increase is mainly attributed to the extension of life expectancy. The risk of developing dementia grows with increasing age, for 65–69-year-olds the disease will annually occur in 2.4 per 1000 persons, for 90+ year-olds, the incidence rate is 70.2 per 1000 persons.^48^

Dementia has no curative or course-altering treatment, preventive measures and early diagnosis are therefore vital^49^ and should aim to influence modifiable risk factors; such as cardiovascular and lifestyle factors, depression, and head injuries.^45^^,^^50^

## 4 Brain-stomatognathic axis

The neurological impact of oral health has received little attention. However, oral health and the brain may influence the development of several diseases, reflecting interesting directions of research and potential management methods. A complex communication system between the brain, and the stomatognathic system – consisting of the jaws, the oral cavity's interior, and surrounding tissues, constitute the brain-stomatognathic axis.^51^ This system explains the relationship between observable changes in the brain and oral status.^51^^,^^52^

The colloquially known “Nun study”, began in 1986 and is still ongoing, with a focus on aging and Alzheimer's disease. 678 women with a mean age of 83 years, agreed to receive cognitive assessments annually, and brain donation on death. Additionally, several of these individuals have dental data.^53^ The study has shown that the number of missing teeth was associated with an increased risk of dementia,^53^ highlighting the association between cognitive decline and masticatory dysfunction, supported by clinical^54^ and animal observations.^55^ These observations suggest tooth loss, is a possible risk factor for cognitive decline^54^, ^55^, ^56^, ^57^, ^58^, ^59^ and dementia.^57^^,^^58^^,^^60^ 7.6% of the global population is edentulous, the prevalence increases to 14% in 50+ year-olds.^61^ Edentulism and tooth loss lead to diminished mastication, so-called masticatory dysfunction.^62^ As the Nun study sparked interest in the association of dementia and tooth loss and the brain-stomatognathic, it may imply that rehabilitating one's masticatory function can prevent cognitive decline.^51^

Rehabilitation of masticatory dysfunction may be achievable by prosthodontic means, such as dentures or dental implants, or by exercise. Both approaches show positive findings. The effects on cognitive function with masticatory exercise intervention exhibit promising results^63^, ^64^, ^65^, ^66^, ^67^ Jaw-tapping, a form of masticatory exercise, for four weeks improves memory function in cognitively impaired patients.^63^ The oral rehabilitation of four edentulous patients, in the case of converting from removable dentures to implant-retained dentures, resulted in improved working memory and oral health quality of life. The pilot investigation suggested that oral rehabilitation influences neurocognitive changes positively.^64^ Evidence suggests that masticatory function has a positive influence on cognitive function.^51^^,^^60^^,^^63^, ^64^, ^65^, ^66^, ^67^

Animal studies shed light on possible mechanisms explaining the influence of mastication,^59^^,^^68^, ^69^, ^70^, ^71^, ^72^, ^73^, ^74^ and involve cognitive decline as a result of decreased cellular proliferation^70^^,^^71^ and brain-derived neurotrophic factor,^68^ increased nitrous oxide^75^ and extracellular dopamine concentrations^76^ in the hippocampus. These changes have been suggested to be regulated by the cerebellum or movement-compensation in the brain, or sensory-feedback mechanisms pertaining to the stomatognathic complex.^51^ However, caution must be exercised as other factors may influence these relationships. For example, the chronological order of whether cognitive impairment favors poor oral health and thus tooth loss and reduced function of mastication, or if masticatory dysfunction leads to cognitive decline, is challenging to prove.^56^ In addition, the role of another cause of tooth loss, specifically periodontitis, needs to be taken into consideration.^77^

## 5 Relationship between oral health and mental health

Dental caries, severe periodontitis, and tooth loss^26^ are the main oral diseases that remain untreated among 3.5 billion individuals, which reflects oral health as a globally underestimated and undervalued health challenge.^78^^,^^79^ In 2017, the global burden of all oral diseases reached 18.3 million years lived with disability (disability-adjusted life-years for oral conditions), representing a 19.9% increase since 1990,^80^ whereas the global economic burden is 544 billion USD, 187 billion of these are due to productivity losses.^81^

**Fig. 1:**
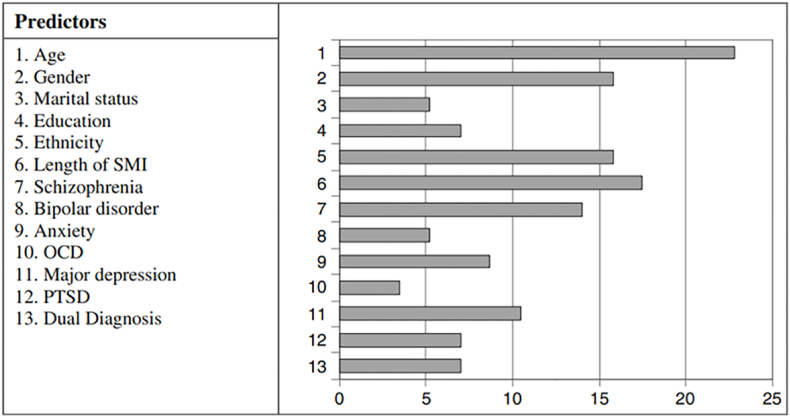
An overview of predictors of poor oral health. Adapted with permission from Kenny et al..^27^

**Fig. 2:**
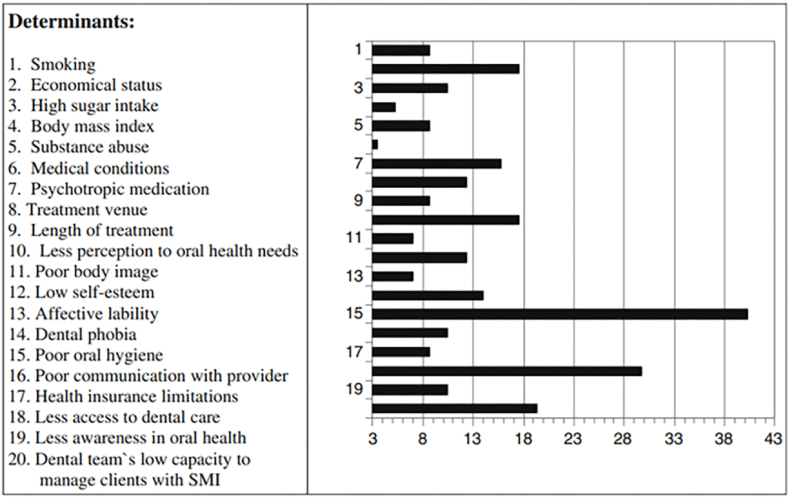
Overview of determinants of poor oral health. Adapted with permission from Kenny et al..^27^

Indirect costs, such as productivity losses, may be attributed to oral health's influence on social withdrawal and isolation, pain and reduced mastication, self-esteem, lack of oral health awareness, and mistrust of dental health care providers.^82^ Individuals with mental disorders may be considered a vulnerable group because of those influences.^82^^,^^83^ Additionally, oral health impacts general health, evidence supports an intimate association with coronary and respiratory diseases, stroke, and diabetes.^84^, ^85^, ^86^, ^87^ Such conditions are frequent comorbidities in those suffering from mental disorders.^14^, ^88^ Studies report plenty of associations between oral and mental health problems: dental erosion in the eating disorders anorexia and bulimia, burning mouth syndrome in anxiety and depression,^89^ dental caries due to high consumption of sugar,^90^ and increased incidence of periodontal disease due to poor hygiene and excessive smoking,^14^, ^82^, ^91^ and increased risk of temporomandibular joint dysfunction.^92^ Reportedly, serious mental disorders involve 2.8 times higher risk of becoming edentulous in contrast to the general population.^14^, ^27^ Predictors and determinants of poor oral health involve mental disorders and lifestyle factors, illustrating the complicated interplay of influencing factors in mental and oral health (Fig. 1, Fig. 2).

Suspicion of mental disorders should be raised at the dental office when atypical presentations of common oral diseases, or uncommon oral diseases, are observed. Common conditions such as loss of surface tooth substance may be attributed to a number of mental disorders based on their representations. Dental erosions are present in 38% of patients with an eating disorder,^93^ the palatal dental surfaces are usually affected because of self-induced vomiting, in contrast to the common labial erosions due to extrinsic consumption of acidic drinks.^94^ Bruxism, colloquially known as teeth grinding, is likely to suffer from anxiety or depression.^95^ Bruxism can result in vertical loss of tooth substance, whereas labial surface loss due to frantic tooth brushing, tends to be linked to obsessive-compulsive disorder.^96^ Xerostomia, the feeling of dry mouth, and hyposalivation are associated with antidepressants and psychotics. For example, tricyclic antidepressants can halve a person's rate of salivation.^97^ A dry mouth increases dental caries risk, weakens denture retention and raises the risk of candida infections.^98^

## 6 Effect of oral health approaches for psycho-disorders

There is a complex relationship between mental disorders and oral diseases due to the shared social determinants and bidirectional interaction mechanisms that involve interconnected social, behavioral, psychological, and biological processes.^99^ Poor oral health has an effect on daily functioning and quality of life especially in patients with mental health disorders.^100^, ^101^, ^102^ Therefore, patients with mental disorders are three times more likely to lose their teeth compared to general people.^103^ There is a burden of oral health-related quality of life in patients with a mental health disorder. It is important to develop an oral health toolkit considering its potential effect on oral health-related quality of life in mental health.^100^ Oral health programs should be provided tailored to the needs of the patient. Effective oral care is necessary for maintaining the oral health of mental health patients.^104^ When caring for patients with mental health disorders, it is essential to consider oral health care as an essential part of their daily tasks and provide necessary nursing support. Mental health nurses have an important role in the care of patients with mental health disorders.^100^ In clinics, mental health nurses including physicians and dental professionals should be involved in the oral health care of mental health disorder patients. Mental health nurses should be more aware of oral health and oral health risk and should provide long-term interventions to improve oral health. Therefore, multidisciplinary teams involved in the care of mental health disorders, and should consider oral health care as an essential part of their care for patients with mental health disorders.^105^

## 7 Mental disorders and dental diseases

Periodontitis is a chronic disease, that affects the tissue surrounding the tooth structure, with inflammatory disorder, degradation, and ultimately loss of the tooth. The treatment constitutes the removal of bacterial biofilm on the tooth surface regularly by dental professionals and demands a high level of adherence to excellent oral hygiene routines. Beyond tooth loss and subsequent masticatory dysfunction, periodontitis also affects general health.^84^, ^85^, ^86^^,^^106^^,^^107^ Globally, periodontitis is among the most prevalent diseases with 20–50% of the population affected.^108^ The prevalence of periodontitis is projected to increase with the aging population and as a larger portion of older adults retain their natural teeth.^109^ Mental disorders and their relationship with oral disease, in particular periodontitis, are gaining increasingly more attention in the research communities. Several of the relationships are suggested to be bi-directional, which opens up for future therapeutic, diagnostic, and preventive measures.^110^

### 7.1 Alzheimer's disease

Recent meta-analyses suggest a significant association between periodontitis and Alzheimer's disease.^77^^,^^111^ Indeed, a number of studies support this association and propose several explanations,^112^^,^^113^ however, the mechanism of bacterial translocation contributing to systemic inflammation, seems plausible.^110^ This is supported as the DNA of *Porphyromonas gingivalis*, one of the main pathogens of periodontitis has been isolated in Alzheimer-diagnosed individuals,^114^ as well as antibodies against several periodontitis-associated bacteria.^115^^,^^116^ Additionally, an association between periodontitis severity and Alzheimer's disease has been reported.^111^^,^^117^^,^^118^ Further support for this evidence is attributed to animal studies where murine subjects were administered live *P. gingivalis* or their endotoxin, lipopolysaccharide. The administrations resulted in observable reductions of learning and memory functions, and plaques of amyloid-β, a typical histological finding associated with Alzheimer's disease, in the animals' hippocampus.^119^, ^120^, ^121^ Consequently, these findings have inspired the development of gingipain inhibitors, which inhibit *P. gingivalis*' proteases. The inhibitors resulted in decreased plaque formation, bacterial volumes, and protective effects on hippocampus cells.^114^

Taken together, the literature suggests periodontitis to be a modifiable risk factor for dementia, particularly Alzheimer's disease, and can therefore be an aim for therapeutic and prophylactic measures.^122^

### 7.2 Depression

The role of bacteria may also play a role in the relationship between periodontitis and depression. Indeed, significant differences in the oral microbiome in depressed individuals have been reported. 21 taxa differed in abundance between the healthy and depressed subjects, and elevated levels of *Neisseria* spp. and *Prevotella nigrescens* were noted (REF: Variations in the oral microbiome are associated with depression in young adults.^123^ Animal studies support the notion that oral administration of lipopolysaccharide or live periodontitis-associated pathogens results in elevated inflammatory markers systemically, including the brain, and depressive-like behavior.^124^, ^125^, ^126^ A genetic relationship has been suggested. An elegant study investigated the role of crosstalk genes and neuropeptides in these two diseases. The neuropeptides adrenomedullin, insulin-like growth factor 2, prodynorphin, and resistin were identified as mutually expressed in both periodontitis and depression, also playing a role in identifying depression.^127^

Epidemiological data reports a 62.5% comorbidity rate of depression among periodontitis patients, compared to healthy individuals (38.86%).^128^ Another study, for 10 years, followed over 60,000 subjects and reported elevated depression incidence among the ones with periodontitis.^129^ Depression is influenced by psychological and social factors. Periodontitis symptoms, such as malodor, poor oral hygiene, and edentulousness may negatively influence psychosocial factors by favoring social isolation, shame, and reduced self-esteem, and thus contribute to depression.^130^^,^^131^ When a tooth is lost, it may be replaced by a dental implant, however, an implant may also develop peri-implantitis, similar to periodontitis. Ultimately, the implant may be lost. Several of the effects and disease relationships may be extrapolated from periodontitis to peri-implantitis.^132^ Periodontitis and peri-implantitis may be suggestive modifiable risk factors for depression, and if so, simple periodontal intervention and oral hygiene instruction may prevent or aid in the treatment of depression.^133^

### 7.3 Bipolar disorder

Chronic inflammation has been reported as a factor in bipolar disorder. The relationship between bipolar disorder and periodontitis was examined from 2001 to 2012 in Taiwan. For bipolar disorder, an elevated risk was reported among the periodontitis subjects, compared to the non-periodontitis subjects.^134^ Higher bacterial loads of periodontitis-associated pathogens were reported in subjects with periodontitis and bipolar disorder, compared to patients with periodontitis but mentally healthy.^135^ These studies support a possible relationship between bipolar disorder and periodontitis, which warrants further investigations.

### 7.4 Parkinson's disease

The biological relationship between Parkinson's disease and periodontitis is less understood. However, some studies do report an elevated prevalence of periodontitis among Parkinson's patients.^136^ Parkinson's disease causes motor disruption and cognitive impairment resulting from neuronal cell death in the brain's dopamine-producing neurons of the substantia nigra.^137^ Hand tremors and rigidity are common symptoms, which make it challenging to maintain adequate daily oral hygiene. The disease itself can arguably be a risk factor for periodontitis, however, epidemiological evidence supports that periodontitis increases the risk of Parkinson's disease.^138^^,^^139^ A protective effect against Parkinson's disease has been observed in patients receiving periodontal treatment over 5 consecutive years, compared to patients who did not receive treatment at all or for 5 consecutive years.^138^^,^^139^ Authors speculate that inflammation initiated and maintained by periodontitis-associated pathogens entering the brain ultimately contributes to or causes Parkinson's disease.^140^ Further research to elaborate mechanistic relationships and the influence of periodontal treatment on established Parkinson's disease may be interesting for future directions.

### 7.5 Schizophrenia

Supportive literature on the relationship between periodontitis and schizophrenia is scarce. A few studies have reported that patients with schizophrenia carry an elevated risk of periodontitis and an even higher risk for those consuming antipsychotics.^141^ Cortisol, commonly implicated in periodontitis, has been ruled out as the levels were lower among schizophrenia patients compared to healthy ones.^142^ Interestingly, the angiotensin-converting enzyme gene's D allele is reportedly a protective factor against schizophrenia^143^ and periodontitis,^144^ and may prove to be a biological connection. In addition, the role of the oropharyngeal microbiome, the salivary microbiome, and periodontitis was suggested to potentially be associated with schizophrenia, and that periodontitis reinforces the role of inflammation in the pathophysiology of schizophrenia. Moreover, saliva is a bodily fluid of diagnostic interest for several conditions,^145^ and may be useful in the diagnostics of schizophrenia as well, however, the current evidence is still limited.^146^

## 8 Social media influence oral health and mental health

The impact of social media on mental health,^20^, ^21^, ^22^, ^23^ represents another mechanism of the mental health-oral health relationship. Social media constitute an array of web-based services allowing users to interact both verbally and visually.^147^ Social media use is particularly widespread among teenagers between 13 and 17 years old.^148^^,^^149^ This demographic is worrying as accumulating evidence supports social media's negative influence on mental health, as research suggests that half of the mental disorders are formed by the age of 14 and 75% by 18 years old.^150^^,^^151^ A recent systematic review highlighted that depression, anxiety, and psychological distress in adolescents correlated with time spent, activity, investment, and addiction to social media.^152^

Dental practitioners enjoy posting cases on social media,^153^^,^^154^ this may be interesting for dental professionals, however, the impact of such posts has shown an inclination among patients to seek cosmetic modifications for their smiles.^155^ Indeed, exposure to “ideal” facial pictures, increased smiles, and face dissatisfaction among young adults.^156^ Smile dissatisfaction and self-perceived need for dental makeovers affect mental well-being,^157^^,^^158^ and may lead to reduced social function and negative coping strategies; such as abstaining from showing teeth during laughing, eating, and in social settings.^157^^,^^159^^,^^160^ A severe engrossment of a self-recognized defect in appearance is known as body dysmorphic disorder. The engrossment is exaggerated as others do not notice the defect.^161^ Individuals affected by body dysmorphic disorder may present in dental practices, influenced by social media, seeking treatment that is unrealistic and not needed. Dental practitioners need to recognize the role of social media in treatment-seeking and to carefully evaluate the patient's request with one's clinical judgment.^25^

## 9 Management of mental disorders

Several mental disorders are associated with oral diseases, specifically periodontitis, proposing a possible bi-directional relationship. Treatment and prevention of periodontitis may yield protective effects against several mental disorders.^110^^,^^112^^,^^139^^,^^162^ However, the management of patients with mental disorders is multidimensional and highly dynamic, needing individual assessments and management modifications from patient to patient and from appointment to appointment,^27^ making them a challenging patient group. However, in general, patients with mental health disorders should receive thorough oral and periodontal health information, hygiene instruction, education, and regular follow-ups, to improve patient's awareness, habits, and literacy.^91^^,^^163^ Multidisciplinary interventions may further improve compliance, dental fear, oral health, and habits, and contribute to a more positive prognosis.^132^^,^^164^ Dental practitioners should receive education on mental disorders, to better manage, communicate with, and identify these patients, as well as to cooperate with other health professionals. Dental health care should be integrated with existing psychiatric rehabilitation and preventive programs.^82^ Other health professionals should be educated on the impact of oral health on mental health and be aware of the need for dental services to achieve a fully and easily accessible multidisciplinary program taking the whole individual into consideration.

## 10 Future directions

Several proposed explanations for the interplay of oral health and mental disorders involve dysregulated microbiomes, translocated bacteria, and systemic inflammation, among others. Future investigations should strive to elucidate the exact biological relationships, to develop new directions for treatment. An example is the gingipain inhibitor, COR388, for the treatment of Alzheimer's disease, currently in a phase 2/3 clinical trial.^114^^,^^165^ Potential protective effects of periodontal treatment should be investigated by longitudinal studies with sizable populations for similar effects.^132^ The therapeutic potential of oral health interventions on mental disorders is a little-researched area that deserves further investigation. For patients with mental disorders, studies should investigate the effect of, and obstacles in, multidisciplinary interventions and preventive programs to guide management recommendations and guidelines.

## 11 Conclusion

Mental disorders are indeed an expanding threat, which requires raised awareness, education, prevention, and treatment initiatives nationally and globally. There is a complex relationship between mental disorders and oral diseases. Various oral health problems are associated with mental health problems. The interplay of oral health and mental disorders involves dysregulated microbiome, translocated bacteria, and systemic inflammation, among others. Mental health nurses including physicians and dental professionals should be involved in the oral health care of mental health disorder patients. Therefore, multidisciplinary should be involved in the care of mental health disorders, and they should consider oral health care as an essential part of their care for patients with mental health disorders. Future investigations should strive to elucidate the exact biological relationships, to develop new directions for treatment.

## References

[1] Søvold L.E., Naslund J.A., Kousoulis A.A. (2021). Prioritizing the mental health and well-being of healthcare workers: an urgent global public health priority. Front Public Health.

[2] Santomauro D.F., Herrera A.M.M., Shadid J. (2021). Global prevalence and burden of depressive and anxiety disorders in 204 countries and territories in 2020 due to the COVID-19 pandemic. Lancet.

[3] Tribst J.P.M., Dal Piva AMdO., Madruga C.F.L. (2018). Endocrown restorations: influence of dental remnant and restorative material on stress distribution. Dent Mater.

[4] (2013). Diagnostic and Statistical Manual of Mental Disorders: DSM-5.

[5] Nochaiwong S., Ruengorn C., Thavorn K. (2021). Global prevalence of mental health issues among the general population during the coronavirus disease-2019 pandemic: a systematic review and meta-analysis. Sci Rep.

[6] (2022). COVID-19 pandemic triggers 25% increase in prevalence of anxiety and depression worldwide. https://www.who.int/news/item/02-03-2022-covid-19-pandemic-triggers-25-increase-in-prevalence-of-anxiety-and-depression-worldwide.

[7] Petersen P.E., Kwan S. (2011). Equity, social determinants and public health programmes–the case of oral health. Community Dent Oral Epidemiol.

[8] Marmot M., Bell R. (2011). Social determinants and dental health. Adv Dent Res.

[9] Arora T., Grey I. (2020). Health behaviour changes during COVID-19 and the potential consequences: a mini-review. J Health Psychol.

[10] Kisely S. (2016). No mental health without oral health. Can J Psychiatr.

[11] Ramon T., Grinshpoon A., Zusman S., Weizman A. (2003). Oral health and treatment needs of institutionalized chronic psychiatric patients in Israel. Eur Psychiatr.

[12] Fratto G., Manzon L. (2014). Use of psychotropic drugs and associated dental diseases. Int J Psychiatr Med.

[13] Wey M.C., Loh S., Doss J.G., Abu Bakar A.K., Kisely S. (2016). The oral health of people with chronic schizophrenia: a neglected public health burden. Aust N Z J Psychiatr.

[14] Kisely S., Baghaie H., Lalloo R., Siskind D., Johnson N.W. (2015). A systematic review and meta-analysis of the association between poor oral health and severe mental illness. Psychosom Med.

[15] Torales J., O'Higgins M., Castaldelli-Maia J.M., Ventriglio A. (2020). The outbreak of COVID-19 coronavirus and its impact on global mental health. Int J Soc Psychiatr.

[16] Gustafsson A., Persson C., Källestål C. (2020). Predicting non-attendance: a model of the complex relationships in dental care non-attendance among adolescents in örebro county, Sweden. Psychology.

[17] Stein Duker L.I., Grager M., Giffin W., Hikita N., Polido J.C. (2022). The relationship between dental fear and anxiety, general anxiety/fear, sensory over-responsivity, and oral health behaviors and outcomes: a conceptual model. Int J Environ Res Publ Health.

[18] Nazir M., Almulhim K.S., AlDaamah Z. (2021). Dental fear and patient preference for emergency dental treatment among adults in COVID-19 quarantine centers in dammam, Saudi arabia. Patient Prefer Adherence.

[19] Daly J., Black E.A. (2020). The impact of COVID-19 on population oral health. Community Dent Health.

[20] Brailovskaia J., Margraf J. (2021). The relationship between burden caused by coronavirus (Covid-19), addictive social media use, sense of control and anxiety. Comput Hum Behav.

[21] Cinelli M., Quattrociocchi W., Galeazzi A. (2020). The COVID-19 social media infodemic. Sci Rep.

[22] Drouin M., McDaniel B.T., Pater J., Toscos T. (2020). How parents and their children used social media and technology at the beginning of the COVID-19 pandemic and associations with anxiety. Cyberpsychol, Behav Soc Netw.

[23] Hou F., Bi F., Jiao R., Luo D., Song K. (2020). Gender differences of depression and anxiety among social media users during the COVID-19 outbreak in China:a cross-sectional study. BMC Publ Health.

[24] Dias da Silva M.A., Walmsley A.D. (2019). Fake news and dental education. Br Dent J.

[25] Rana S., Kelleher M. (2018). The dangers of social media and young dental patients' body image. Dent Update.

[26] Peres M.A., Macpherson L.M., Weyant R.J. (2019). Oral diseases: a global public health challenge. Lancet.

[27] Kenny A., Dickson-Swift V., Gussy M. (2020). Oral health interventions for people living with mental disorders: protocol for a realist systematic review. Int J Ment Health Syst.

[28] Dattani S., Ritchie H., Roser M. (2021). https://ourworldindata.org/mental-health.

[29] Silva S.A., Silva S.U., Ronca D.B., Gonçalves V.S.S., Dutra E.S., Carvalho K.M.B. (2020). Common mental disorders prevalence in adolescents: a systematic review and meta-analyses. PLoS One.

[30] Trautmann S., Rehm J., Wittchen H.-U. (2016). The economic costs of mental disorders: do our societies react appropriately to the burden of mental disorders?. EMBO Rep.

[31] Collaborators G.M.D. (2022). Global, regional, and national burden of 12 mental disorders in 204 countries and territories, 1990–2019: a systematic analysis for the Global Burden of Disease Study 2019. Lancet Psychiatr.

[32] Bloom D.E., Cafiero E., Jané-Llopis E. (2011).

[33] Gustavsson A., Svensson M., Jacobi F. (2012). Cost of disorders of the brain in Europe 2010. Eur Neuropsychopharmacol.

[34] Bains N., Abdijadid S. (2022).

[35] Orsolini L., Latini R., Pompili M. (2020). Understanding the complex of suicide in depression: from research to clinics. Psychiatry Investig.

[36] Penninx B.W., Pine D.S., Holmes E.A., Reif A. (2021). Anxiety disorders. Lancet.

[37] Chand S.P., Marwaha R., Bender R.M. (2022).

[38] Appukuttan D.P. (2016). Strategies to manage patients with dental anxiety and dental phobia: literature review. Clin Cosmet Invest Dent.

[39] Grisolia B.M., Dos Santos A.P.P., Dhyppolito I.M., Buchanan H., Hill K., Oliveira B.H. (2021). Prevalence of dental anxiety in children and adolescents globally: a systematic review with meta‐analyses. Int J Paediatr Dent.

[40] Hill K., Chadwick B., Freeman R., O'sullivan I., Murray J. (2013). Adult Dental Health Survey 2009: relationships between dental attendance patterns, oral health behaviour and the current barriers to dental care. Br Dent J.

[41] Hare J., Bruj-Milasan G., Newton T. (2019). An overview of dental anxiety and the non-pharmacological management of dental anxiety. Prim Dent J.

[42] Jain A., Mitra P. (2021).

[43] Hany M.R.B., Azhar Y. (2021). Schizophrenia. https://www.ncbi.nlm.nih.gov/books/NBK539864/.

[44] Knopman D.S., Petersen R.C. (2014). Mild cognitive impairment and mild dementia: a clinical perspective. Mayo Clin Proc.

[45] Han J.Y., Han S.H. (2014). Primary prevention of Alzheimer's disease: is it an attainable goal?. J Kor Med Sci.

[46] Stephan Y., Sutin A.R., Luchetti M., Terracciano A. (2018). Subjective age and risk of incident dementia: evidence from the national health and aging trends survey. J Psychiatr Res.

[47] Collaborators G.D.F. (2022). Estimation of the global prevalence of dementia in 2019 and forecasted prevalence in 2050: an analysis for the Global Burden of Disease Study 2019. Lancet Public Health.

[48] van der Flier W.M., Scheltens P. (2005). Epidemiology and risk factors of dementia. J Neurol Neurosurg Psychiatry.

[49] Fayosse A., Nguyen D.-P., Dugravot A. (2020). Risk prediction models for dementia: role of age and cardiometabolic risk factors. BMC Med.

[50] Tariq S., Barber P.A. (2018). Dementia risk and prevention by targeting modifiable vascular risk factors. J Neurochem.

[51] Lin C.S. (2018). Revisiting the link between cognitive decline and masticatory dysfunction. BMC Geriatr.

[52] Lin C.S., Yeung A.W.K. (2020). What do we learn from brain imaging?-A primer for the dentists who want to know more about the association between the brain and human stomatognathic functions. J Oral Rehabil.

[53] Stein P.S., Desrosiers M., Donegan S.J., Yepes J.F., Kryscio R.J. (2007). Tooth loss, dementia and neuropathology in the Nun study. J Am Dent Assoc.

[54] Okamoto N., Morikawa M., Amano N., Yanagi M., Takasawa S., Kurumatani N. (2017). Effects of tooth loss and the apolipoprotein E ϵ4 allele on mild memory impairment in the Fujiwara-kyo study of Japan: a nested case-control study. J Alzheim Dis.

[55] Chen H., Iinuma M., Onozuka M., Kubo K.-Y. (2015). Chewing maintains hippocampus-dependent cognitive function. Int J Med Sci.

[56] Tada A., Miura H. (2017). Association between mastication and cognitive status: a systematic review. Arch Gerontol Geriatr.

[57] Cerutti-Kopplin D., Feine J., Padilha D. (2016). Tooth loss increases the risk of diminished cognitive function: a systematic review and meta-analysis. JDR Clin Trans Res.

[58] Luo J., Wu B., Zhao Q. (2015). Association between tooth loss and cognitive function among 3063 Chinese older adults: a community-based study. PLoS One.

[59] Avivi-Arber L., Seltzer Ze, Friedel M. (2017). Widespread volumetric brain changes following tooth loss in female mice. Front Neuroanat.

[60] Suma S., Furuta M., Yamashita Y., Matsushita K. (2019). Aging, Mastication, and Malnutrition and Their associations with cognitive disorder: evidence from epidemiological data. Curr Oral Health Rep.

[61] Tyrovolas S., Koyanagi A., Panagiotakos D.B. (2016). Population prevalence of edentulism and its association with depression and self-rated health. Sci Rep.

[62] Ikebe K., Matsuda K., Kagawa R. (2012). Masticatory performance in older subjects with varying degrees of tooth loss. J Dent Res.

[63] Cho S.Y., Jahng G.H., Rhee H.Y. (2014). An fMRI study on the effects of jaw-tapping movement on memory function in elderly people with memory disturbances. Eur J Integr Med.

[64] Tan D., Foster S., Korgaonkar M.S., Oxenham V., Whittle T., Klineberg I. (2020). The role of progressive oral implant rehabilitation in mastication, cognition and oral health‐related quality of life outcomes—a pilot to define the protocol. J Oral Rehabil.

[65] Kim J., Park J., Yim J. (2015). The effects of masticatory exercise using a gum on the cognitive function and stress. Int J BioSci Biotechnol.

[66] Kim T.-H. (2021). Effects of masticatory exercise on cognitive function in community-dwelling older adults. Technol Health Care.

[67] Chuhuaicura P., Dias F.J., Arias A., Lezcano M.F., Fuentes R. (2019). Mastication as a protective factor of the cognitive decline in adults: a qualitative systematic review. Int Dent J.

[68] Kida K., Tsuji T., Tanaka S., Kogo M. (2015). Zinc deficiency with reduced mastication impairs spatial memory in young adult mice. Physiol Behav.

[69] Oue H., Miyamoto Y., Okada S. (2013). Tooth loss induces memory impairment and neuronal cell loss in APP transgenic mice. Behav Brain Res.

[70] Nose-Ishibashi K., Watahiki J., Yamada K. (2014). Soft-diet feeding after weaning affects behavior in mice: potential increase in vulnerability to mental disorders. Neuroscience.

[71] Kubo K.Y., Murabayashi C., Kotachi M. (2017). Tooth loss early in life suppresses neurogenesis and synaptophysin expression in the hippocampus and impairs learning in mice. Arch Oral Biol.

[72] Kawahata M., Ono Y., Ohno A., Kawamoto S., Kimoto K., Onozuka M. (2014). Loss of molars early in life develops behavioral lateralization and impairs hippocampus-dependent recognition memory. BMC Neurosci.

[73] Akazawa Y., Kitamura T., Fujihara Y., Yoshimura Y., Mitome M., Hasegawa T. (2013). Forced mastication increases survival of adult neural stem cells in the hippocampal dentate gyrus. Int J Mol Med.

[74] Suzuki A., Iinuma M., Hayashi S., Sato Y., Azuma K., Kubo K.Y. (2016). Maternal chewing during prenatal stress ameliorates stress-induced hypomyelination, synaptic alterations, and learning impairment in mouse offspring. Brain Res.

[75] Pang Q., Hu X., Li X., Zhang J., Jiang Q. (2015). Behavioral impairments and changes of nitric oxide and inducible nitric oxide synthase in the brains of molarless KM mice. Behav Brain Res.

[76] Ono Y., Koizumi S., Onozuka M. (2015). Chewing prevents stress-induced hippocampal LTD formation and anxiety-related behaviors: a possible role of the dopaminergic system. BioMed Res Int.

[77] Kaliamoorthy S., Nagarajan M., Sethuraman V., Jayavel K., Lakshmanan V., Palla S. (2022). Association of Alzheimer's disease and periodontitis-a systematic review and meta-analysis of evidence from observational studies. Med Pharm Rep.

[78] Kassebaum N.J., Smith A.G., Bernabé E. (2017). Global, regional, and national prevalence, incidence, and disability-adjusted life years for oral conditions for 195 countries, 1990–2015: a systematic analysis for the global burden of diseases, injuries, and risk factors. J Dent Res.

[79] Marcenes W., Kassebaum N.J., Bernabé E. (2013). Global burden of oral conditions in 1990-2010: a systematic analysis. J Dent Res.

[80] Bernabe E., Marcenes W., Hernandez C.R. (2020). Global, regional, and national levels and trends in burden of oral conditions from 1990 to 2017: a systematic analysis for the global burden of disease 2017 study. J Dent Res.

[81] Righolt A.J., Jevdjevic M., Marcenes W., Listl S. (2018). Global-, regional-, and country-level economic impacts of dental diseases in 2015. J Dent Res.

[82] Matevosyan N.R. (2010). Oral health of adults with serious mental illnesses: a review. Community Ment Health J.

[83] Torales J., Barrios I., González I. (2017). Oral and dental health issues in people with mental disorders. Medwave.

[84] Sanz M., Marco del Castillo A., Jepsen S. (2020). Periodontitis and cardiovascular diseases: consensus report. J Clin Periodontol.

[85] Azarpazhooh A., Leake J.L. (2006). Systematic review of the association between respiratory diseases and oral health. J Periodontol.

[86] Llambés F., Arias-Herrera S., Caffesse R. (2015). Relationship between diabetes and periodontal infection. World J Diabetes.

[87] Cabanillas‐Balsera D., Martín‐González J., Montero‐Miralles P., Sánchez‐Domínguez B., Jiménez‐Sánchez M., Segura‐Egea J. (2019). Association between diabetes and nonretention of root filled teeth: a systematic review and meta‐analysis. Int Endod J.

[88] Leng W.D., Zeng X.T., Kwong J.S., Hua X.P. (2015). Periodontal disease and risk of coronary heart disease: an updated meta-analysis of prospective cohort studies. Int J Cardiol.

[89] Robb N., Smith B., Geidrys-Leeper E. (1995). The distribution of erosion in the dentitions of patients with eating disorders. Br Dent J.

[90] DeBate R.D., Plichta S.B., Tedesco L.A., Kerschbaum W.E. (2006). Integration of oral health care and mental health services: dental hygienists' readiness and capacity for secondary prevention of eating disorders. J Behav Health Serv Res.

[91] Khokhar M.A., Khokhar W.A., Clifton A.V., Tosh G.E. (2016). Oral health education (advice and training) for people with serious mental illness. Cochrane Database Syst Rev.

[92] Tiwari T., Kelly A., Randall C.L., Tranby E., Franstve-Hawley J. (2021). Association between mental health and oral health status and care utilization. Front Oral Health.

[93] Ahmed K.E. (2013). The psychology of tooth wear. Spec Care Dent.

[94] Milosevic A. (1999). Eating disorders and the dentist. Br Dent J.

[95] Sutin A.R., Terracciano A., Ferrucci L., Costa P.T. (2010). Teeth grinding: is emotional stability related to bruxism?. J Res Pers.

[96] Ghanizadeh A. (2008). ADHD, bruxism and psychiatric disorders: does bruxism increase the chance of a comorbid psychiatric disorder in children with ADHD and their parents?. Sleep & breathing = Schlaf & Atmung.

[97] Daly C. (2016). Oral and dental effects of antidepressants. Aust Prescr.

[98] Barbe A.G. (2018). Medication-induced xerostomia and hyposalivation in the elderly: culprits, complications, and management. Drugs Aging.

[99] Joury E., Kisely S., Watt R.G. (2023). Mental disorders and oral diseases: future research directions. J Dent Res.

[100] Kuipers S., Boonstra N., Kronenberg L., Keuning-Plantinga A., Castelein S. (2021). Oral health interventions in patients with a mental health disorder: a scoping review with critical appraisal of the literature. Int J Environ Res Publ Health.

[101] Petersen P.E. (2003). The world oral health report 2003: continuous improvement of oral health in the 21st century--the approach of the WHO global oral health programme. Community Dent Oral Epidemiol.

[102] Petersen P.E. (2005). Priorities for research for oral health in the 21st century--the approach of the WHO Global Oral Health Programme. Community Dent Health.

[103] Kisely S., Baghaie H., Lalloo R., Siskind D., Johnson N.W. (2015). A systematic review and meta-analysis of the association between poor oral health and severe mental illness. Psychosom Med.

[104] Dagnew Z.A., Abraham I.A., Beraki G.G., Tesfamariam E.H., Mittler S., Tesfamichael Y.Z. (2020). Nurses' attitude towards oral care and their practicing level for hospitalized patients in Orotta National Referral Hospital, Asmara-Eritrea: a cross-sectional study. BMC Nurs.

[105] Peters M.D., Godfrey C.M., Khalil H., McInerney P., Parker D., Soares C.B. (2015). Guidance for conducting systematic scoping reviews. Int J Evid Base Healthc.

[106] Mehrotra N., Singh S. (2021).

[107] Nagpal R., Yamashiro Y., Izumi Y. (2015). The two-way association of periodontal infection with systemic disorders: an overview. Mediat Inflamm.

[108] Nazir M., Al-Ansari A., Al-Khalifa K., Alhareky M., Gaffar B., Almas K. (2020). Global prevalence of periodontal disease and lack of its surveillance. Sci World J.

[109] Listl S., Galloway J., Mossey P., Marcenes W. (2015). Global economic impact of dental diseases. J Dent Res.

[110] Hashioka S., Inoue K., Miyaoka T. (2019). The possible causal link of periodontitis to neuropsychiatric disorders: more than psychosocial mechanisms. Int J Mol Sci.

[111] Leira Y., Domínguez C., Seoane J. (2017). Is periodontal disease associated with Alzheimer's disease? A systematic review with meta-analysis. Neuroepidemiology.

[112] Chen C.K., Wu Y.T., Chang Y.C. (2017). Association between chronic periodontitis and the risk of Alzheimer's disease: a retrospective, population-based, matched-cohort study. Alzheimer's Res Ther.

[113] Choi S., Kim K., Chang J. (2019). Association of chronic periodontitis on Alzheimer's disease or vascular dementia. J Am Geriatr Soc.

[114] Dominy S.S., Lynch C., Ermini F. (2019). Porphyromonas gingivalis in Alzheimer's disease brains: evidence for disease causation and treatment with small-molecule inhibitors. Sci Adv.

[115] Kamer A.R., Craig R.G., Pirraglia E. (2009). TNF-alpha and antibodies to periodontal bacteria discriminate between Alzheimer's disease patients and normal subjects. J Neuroimmunol.

[116] Cestari J.A., Fabri G.M., Kalil J. (2016). Oral infections and cytokine levels in patients with Alzheimer's disease and mild cognitive impairment compared with controls. J Alzheimers Dis.

[117] Noble J.M., Scarmeas N., Celenti R.S. (2014). Serum IgG antibody levels to periodontal microbiota are associated with incident Alzheimer disease. PLoS One.

[118] Holmer J., Eriksdotter M., Schultzberg M., Pussinen P.J., Buhlin K. (2018). Association between periodontitis and risk of Alzheimer's disease, mild cognitive impairment and subjective cognitive decline: a case-control study. J Clin Periodontol.

[119] Ding Y., Ren J., Yu H., Yu W., Zhou Y. (2018). Porphyromonas gingivalis, a periodontitis causing bacterium, induces memory impairment and age-dependent neuroinflammation in mice. Immun Ageing.

[120] Ilievski V., Zuchowska P.K., Green S.J. (2018). Chronic oral application of a periodontal pathogen results in brain inflammation, neurodegeneration and amyloid beta production in wild type mice. PLoS One.

[121] Zhang J., Yu C., Zhang X. (2018). Porphyromonas gingivalis lipopolysaccharide induces cognitive dysfunction, mediated by neuronal inflammation via activation of the TLR4 signaling pathway in C57BL/6 mice. J Neuroinflammation.

[122] Lee Y.T., Lee H.C., Hu C.J. (2017). Periodontitis as a modifiable risk factor for dementia: a nationwide population-based cohort study. J Am Geriatr Soc.

[123] Wingfield B., Lapsley C., McDowell A. (2021). Variations in the oral microbiome are associated with depression in young adults. Sci Rep.

[124] Norden D.M., Trojanowski P.J., Villanueva E., Navarro E., Godbout J.P. (2016). Sequential activation of microglia and astrocyte cytokine expression precedes increased Iba-1 or GFAP immunoreactivity following systemic immune challenge. Glia.

[125] Townsend B.E., Chen Y.J., Jeffery E.H., Johnson R.W. (2014). Dietary broccoli mildly improves neuroinflammation in aged mice but does not reduce lipopolysaccharide-induced sickness behavior. Nutr Res.

[126] Martínez M., Martín‐Hernández D., Virto L. (2021). Periodontal diseases and depression: a pre‐clinical in vivo study. J Clin Periodontol.

[127] Sun C., Han J., Bai Y., Zhong Z., Song Y., Sun Y. (2021). Neuropeptides as the shared genetic crosstalks linking periodontitis and major depression disorder. Dis Markers.

[128] Laforgia A., Corsalini M., Stefanachi G., Pettini F., Di Venere D. (2015). Assessment of psychopatologic traits in a group of patients with adult chronic periodontitis: study on 108 cases and analysis of compliance during and after periodontal treatment. Int J Med Sci.

[129] Hsu C.C., Hsu Y.C., Chen H.J. (2015). Association of periodontitis and subsequent depression: a nationwide population-based study. Medicine.

[130] Saintrain M.V., de Souza E.H. (2012). Impact of tooth loss on the quality of life. Gerodontology.

[131] Dumitrescu A.L. (2016). Depression and inflammatory periodontal disease considerations-an interdisciplinary approach. Front Psychol.

[132] D'Ambrosio F., Caggiano M., Schiavo L. (2022). Chronic stress and depression in periodontitis and peri-implantitis: a narrative review on neurobiological, neurobehavioral and immune-microbiome interplays and clinical management implications. Dent J.

[133] Hashioka S., Inoue K., Hayashida M., Wake R., Oh-Nishi A., Miyaoka T. (2018). Implications of systemic inflammation and periodontitis for major depression. Front Neurosci.

[134] Huang Y.-K., Wang Y.H., Chang Y.C. (2020). Chronic periodontitis is associated with the risk of bipolar disorder: a population-based cohort study. Int J Environ Res Publ Health.

[135] Cunha F.A., Cota L.O., Cortelli S.C. (2019). Periodontal condition and levels of bacteria associated with periodontitis in individuals with bipolar affective disorders: a case‐control study. J Periodontal Res.

[136] Schwarz J., Heimhilger E., Storch A. (2006). Increased periodontal pathology in Parkinson's disease. J Neurol.

[137] Zafar S., Yaddanapudi S.S. (2021).

[138] Chen C.K., Huang J.Y., Wu Y.T., Chang Y.C. (2018). Dental scaling decreases the risk of Parkinson's disease: a nationwide population-based nested case-control study. Int J Environ Res Publ Health.

[139] Chen C.K., Wu Y.T., Chang Y.C. (2017). Periodontal inflammatory disease is associated with the risk of Parkinson's disease: a population-based retrospective matched-cohort study. PeerJ.

[140] Bian M., Chen L., Lei L. (2021). Research progress on the relationship between chronic periodontitis and Parkinson's disease. J Zhejiang Univ Med Sci.

[141] Hu K.F., Ho P.S., Chou Y.H., Tsai J.H., Lin C.H.R., Chuang H.-Y. (2020). Periodontal disease and effects of antipsychotic medications in patients newly diagnosed with schizophrenia: a population-based retrospective cohort. Epidemiol Psychiatr Sci.

[142] Albahli B.F., Alrasheed N.M., Alabdulrazaq R.S., Alasmari D.S., Ahmed M.M. (2021). Association between schizophrenia and periodontal disease in relation to cortisol levels: an ELISA-based descriptive analysis. Egypt J Neurol Psychiatr Neurosurg.

[143] Crescenti A., Gassó P., Mas S. (2009). Insertion/deletion polymorphism of the angiotensin-converting enzyme gene is associated with schizophrenia in a Spanish population. Psychiatr Res.

[144] Gürkan A., Emingil G., Saygan B.H. (2009). Renin-angiotensin gene polymorphisms in relation to severe chronic periodontitis. J Clin Periodontol.

[145] Skallevold H.E., Vallenari E.M., Sapkota D. (2021). Salivary biomarkers in lung cancer. Mediat Inflamm.

[146] Martin S., Foulon A., El Hage W., Dufour-Rainfray D., Denis F. (2022). Is There a link between oropharyngeal microbiome and schizophrenia? A narrative review. Int J Mol Sci.

[147] Carr C.T., Hayes R.A. (2015). Social media: defining, developing, and divining. Atl J Commun.

[148] Lenhart A. (2015). https://www.pewresearch.org/internet/2015/04/09/teens-social-media-technology-2015/.

[149] Lenhart A., Smith A., Anderson M., Duggan M., Perrin A. (2015). https://www.pewresearch.org/internet/2015/08/06/teens-technology-and-friendships/.

[150] Kessler R.C., Amminger G.P., Aguilar‐Gaxiola S., Alonso J., Lee S., Ustun T.B. (2007). Age of onset of mental disorders: a review of recent literature. Curr Opin Psychiatr.

[151] Kim-Cohen J., Caspi A., Moffitt T.E., Harrington H., Milne B.J., Poulton R. (2003). Prior juvenile diagnoses in adults with mental disorder: developmental follow-back of a prospective-longitudinal cohort. Arch Gen Psychiatr.

[152] Keles B., McCrae N., Grealish A. (2020). A systematic review: the influence of social media on depression, anxiety and psychological distress in adolescents. Int J Adolesc Youth.

[153] Grajales F.J., Sheps S., Ho K., Novak-Lauscher H., Eysenbach G. (2014). Social media: a review and tutorial of applications in medicine and health care. J Med Internet Res.

[154] Denecke K., Bamidis P., Bond C. (2015). Ethical issues of social media usage in healthcare. Yearb Med Inform.

[155] Ansari S.H., Alzahrani A.A.A., Abomelha A.M.S., Elhalwagy A.E.A., Alalawi T.N.M., Sadiq T.W.M. (2020). Influence of social media towards the selection of hollywood smile among the university students in Riyadh city. J Fam Med Prim Care.

[156] Sampson A., Jeremiah H.G., Andiappan M., Newton J.T. (2020). The effect of viewing idealised smile images versus nature images via social media on immediate facial satisfaction in young adults: a randomised controlled trial. J Orthod.

[157] Nazir R., Mahmood A., Anwar A. (2014). Assessment of psychosocial implant of dental of aesthetics and self perceived orthodontic treatment need in young adults. Pak Oral Dental J.

[158] AlSagob E.I., Alkeait F., Alhaimy L., Alqahtani M., Hebbal M., Ben Gassem A.A. (2021). Impact of self-perceived dental esthetic on psycho-social well-being and dental self confidence: a cross-sectional study among female students in riyadh city. Patient Prefer Adherence.

[159] Taghavi Bayat J., Hallberg U., Lindblad F., Huggare J., Mohlin B. (2013). Daily life impact of malocclusion in Swedish adolescents: a grounded theory study. Acta Odontol Scand.

[160] Militi A., Sicari F., Portelli M. (2021). Psychological and social effects of oral health and dental aesthetic in adolescence and early adulthood: an observational study. Int J Environ Res Publ Health.

[161] Nicewicz H.R., Boutrouille J.F. (2022).

[162] Gokturk O., Inanir S., Balci Yuce H., Demir O., Aydemir Turkal H. (2021). The effect of periodontal treatment on depression, body image, self esteem and anxiety in individuals: a randomized controlled clinical trial. Ann Med Res.

[163] Macnamara A., Mishu M.P., Faisal M.R., Islam M., Peckham E. (2021). Improving oral health in people with severe mental illness (SMI): a systematic review. PLoS One.

[164] Werner H., Hakeberg M., Dahlström L. (2016). Psychological interventions for poor oral health: a systematic review. J Dent Res.

[165] Zhu C.C., Fu S.Y., Chen Y.X. (2020). Advances in drug therapy for Alzheimer's disease. Curr Med Sci.

